# Pre-Existing Immunity with High Neutralizing Activity to 2009 Pandemic H1N1 Influenza Virus in Shanghai Population

**DOI:** 10.1371/journal.pone.0058810

**Published:** 2013-03-19

**Authors:** Xiaoqing Liu, Yuan Liu, Yanjun Zhang, Zhihui Chen, Ziwei Tang, Qingqiang Xu, Yue Wang, Ping Zhao, Zhongtian Qi

**Affiliations:** 1 Department of Microbiology, Shanghai Key Laboratory of Medical Biodefense, Second Military Medical University, Shanghai, P. R. of China; 2 Department of Infectious Disease, Changhai Hospital, Second Military Medical University, Shanghai, P. R. of China; 3 National Institute for Viral Disease Control and Prevention, China CDC, Beijing, P. R. of China; Centers for Disease Control and Prevention, United States of America

## Abstract

Pre-existing immunity is an important factor countering the pandemic potential of an emerging influenza virus strain. Thus, studying of pre-existing immunity to the 2009 pandemic H1N1 virus (2009 H1N1) will advance our understanding of the pathogenesis and epidemiology of this emerging pathogen. In the present study, sera were collected from 486 individuals in a hospital in Shanghai, China, before the 2009 H1N1 influenza pandemic. The serum anti-hemagglutinins (HA) antibody, hemagglutination inhibition (HI) antibody and neutralizing antibody against the 2009 H1N1 were assayed. Among this population, 84.2%, 14.61% and 26.5% subjects possessed anti-HA antibody, HI antibody and neutralizing antibody, respectively. Although neutralizing antibody only existed in those sera with detectable anti-HA antibody, there was no obvious correlation between the titers of anti-HA and neutralizing antibody. However, the titers of anti-HA and neutralizing antibody against seasonal H1N1 virus were highly correlated. In the same population, there was no correlation between titers of neutralizing antibody against 2009 H1N1 and seasonal H1N1. DNA immunization performed on mice demonstrated that antibodies to the HA of 2009 pandemic and seasonal H1N1 influenza viruses were strain-specific and had no cross-neutralizing activity. In addition, the predicted conserved epitope in the HA of 2009 H1N1 and recently circulating seasonal H1N1 virus, GLFGAIAGFIE, was not an immunologically valid B-cell epitope. The data in this report are valuable for advancing our understanding of 2009 H1N1 influenza virus infection.

## Introduction

Many infectious diseases display epidemic wave patterns due to interaction between pathogen antigens and the pre-existing host immunity barrier [1]–[4]. Indeed, the prevalence of a specific epidemic influenza virus strain decreases significantly when broad immunity is established throughout the host population, and increases when the immune barrier is (or becomes) weak. The novel influenza virus, 2009 H1N1, first emerged in mid-April 2009 and initiated the first influenza pandemic of the 21st century [5]. Despite initial concern that little protective immunity existed in the general population, subsequent epidemiological data showed that morbidity in the elderly was lower than that in younger individuals, suggesting the existence of pre-existing immunity [6]–[13]. Phylogenetic analyses on the HA of the 2009 pandemic H1N1 virus demonstrated its close relationship with the 1918–1919 Spanish H1N1 virus. Molecular analyses showed that the structures of the HAs of both 2009 and 1918 pandemic H1N1 virus strains were very similar [14]. Serological cross-section studies performed on a variety of human populations demonstrated protective immunity in elderly individuals [9], [15].

The current strategy for influenza virus control and prevention is primarily dependent on vaccination along with therapeutic and prophylactic use of neuraminidase inhibitors [16]. Vaccination is a passive response to the emergence of novel influenza virus strains [17], [18]. Thus, improvements in the global anti-influenza strategy are required. Since influenza is so common, understanding the nature of the host immune response to influenza virus is vital. To date, protective immunity to influenza has been regarded as futile, due to the overwhelming number of novel emerging reassorted viruses as well as antigenic shifts and drift in their HA molecules. However, influenza epidemics display a wave pattern, which suggests a contribution of pre-existing immunity [2], [3]. Thus, investigation of protective immunity will advance our understanding of influenza biology and benefit our efforts in influenza control and prevention.

In this study, to evaluate the presence of antibodies against 2009 H1N1 in the general population in Shanghai, China, IgG levels and neutralizing activity against both 2009 pandemic and a seasonal H1N1 virus were assessed in 486 serum samples collected prior to 2009 pandemic influenza outbreak. The mechanism(s) of cross-protection were studied by immunization with homologous and heterologous HA-encoding plasmids in mice model.

## Materials and Methods

### Subjects

In total, 486 serum samples (age ranging from 16 to 88) were obtained from in-and out-patient subjects without influenza virus vaccination history, in Changhai Hospital, Shanghai, China, from December, 2008 to February, 2009. Subjects were considered to have not been exposed to 2009 H1N1 influenza virus because the final sample was taken 3 months prior to the onset of the pandemic. Another 27 samples were taken from 20–22-year-old healthy volunteers inoculated with the 2009 H1N1 inactivated vaccine in December, 2009. All study subjects were selected on the basis of a questionnaire designed to exclude any person with symptoms possibly associated with influenza-like illness in the previous 6 months. All donors gave written informed consent for research use of blood samples. The study design was approved by the Ethical Committee of Second Military Medical University.

### Cells

Madin-Darby canine kidney (MDCK) cells, and human embryonic kidney (HEK) 293T cells were obtained from American Type Culture Collection (Manassas, VA) and grown in Dulbecco's modified essential medium (DMEM; Invitrogen, Carlsbad, CA) supplemented with or without 10% fetal bovine serum.

### Plasmids

The cDNA fragments encoding the full-length HA of A/California/05/2009 (GenBank Accession No. FJ966952) strain and a seasonal H1N1 isolate (similar to the A/Brisbane/59/2007 strain, GenBank Accession No. CY030230.1) were synthesized and inserted into the vector pVRC to produce 2009 HA and seasonal HA expression constructs as we reported previously [15], [19]–[24].

### ELISA

Anti-HA antibody was assayed as described previously [18], [21], [24]. Briefly, ELISA Maxisorp plates (Nunc, Roskilde, Denmark) were coated with 100 µl of 10 µg/ml *Galanthus nivalis* lectin (Sigma, St. Louis, MO) in phosphate-buffered saline (PBS) and incubated overnight at 4°C. Plates were washed with washing buffer (0.05% Tween 20 in PBS), and non-specific binding sites were saturated with BSA buffer (3% BSA and 0.05% Tween 20 in PBS). Crude cell lysates from 2009 HA, seasonal HA expression plasmids, or mock vector transfected HEK 293T cells were added to the plates at 100 µL/well and incubated for 2 h at room temperature (RT). After extensive washing, serial two-fold dilutions of serum in BSA buffer, stating with a 1∶100 dilution, were added (100 µL/well) and incubated for 40 min at RT. The plates were washed, incubated with horseradish peroxidase-conjugated anti-mouse or anti-human IgG (Sigma, USA), diluted 1∶10,000 in BSA buffer, and incubated for 40 min at RT. Plates were again washed, color was developed using 3,3′,5,5′-tetramethylbenzidine (TMB) substrate, and the absorbance at 450 and 630 nm measured. A two-fold increase in mean signal over that in mock-coated wells was considered to be HA antibody-positive. The highest positive dilution was determined to be the antibody titer.

### Preparation of 2009 H1N1 and seasonal H1N1 wild-type virus

2009 pandemic H1N1 and seasonal H1N1 viruses were isolated and confirmed by regular influenza virus survey in Fujian Province. The viruses were grown in chorioallantoic fluid of 10-day-old embryonated chicken eggs and purified by sucrose gradient centrifugation. Virus particles were prepared in PBS, aliquoted, and stored at −80°C until use. The viral titer was determined by titration in MDCK cells and the tissue culture infectious dose affecting 50% of the cells (TCID50) was calculated using the Reed-Muench formula [25].

### Hemagglutination inhibition (HI) assay

HI assays were performed according to standard methods as described recently [24]. To inactivate non-specific inhibitors, sera were treated with receptor destroying enzyme (RDE; Denka Seiken, Tokyo, Japan) overnight at 37°C and then were inactivated by incubation at 56°C for 30 min. RDE-treated sera were two-fold serially diluted in v-bottom microtiter plates, starting at a 1∶10 dilution. An equal volume of virus, adjusted to approximately 4 HA untis/25 µl was added to each well. After 30-min incubation at RT, 50 µl of 0.5% chicken erythrocytes were added to each mixture and incubated at 4°C until a positive hemagglutination reaction developed in the non-serum-containing control wells. The inhibition of hemagglutination at the highest serum dilution was considered as the HI titer of the serum.

### DNA immunization

The 2009 HA and seasonal HA expression plasmids were propagated in *Escherichia coli* XL1-blue and purified using Qiagen Giga columns, according to the manufacturer' instructions. Female, 7-week-old BALB/c mice were purchased from SIPPR-BK Experimental Animal Co. Ltd, Shanghai, China. Mice were injected with 2009 HA or seasonal HA plasmids (100 µg in 100 µL) in both tibialis anterior muscles. Control mice were injected with 100 µg mock vector. Each mouse received a boost immunization 2 weeks later. Sera were collected at 2-week intervals, inactivated at 56°C for 30 min and stored at −80°C until required.

All animal experiments were conducted in accordance to the recommendations in the Guide for the Care and Use of Laboratory Animals of the Second Military Medical University and the Rules for the Medical Laboratory Animal (1998) from Ministry of Health, China. The protocol was approved by the Animal Care and Use Committee of Second Military Medical University.

### Microneutralization (MN) assays

Wild-type 2009 H1N1 or seasonal H1N1 virus (100 TCID50/well) was incubated with twofold serial dilutions of RDE-treated sera. The mixtures were transferred to MDCK target cells in a 96-well plate, incubated at 37°C in the presence of TPCK-trypsin for 2 h. After washing twice, the cells were cultured in medium without TPCK-trypsin to prevent re-entry of viruses. After 24 hours incubation, the cells were detached using 2 mM EDTA in PBS, washed in PBS buffer containing 2% FBS and 0.05% NaN_3_, and incubated with mixed sera of the vaccinated subjects (diluted 1∶100 in the same buffer) for 40 min at RT. Cells were then washed twice with PBS and incubated with FITC-conjugated rabbit anti-human IgG (Jackson ImmunoResearch Laboratories, USA). Fluorescent-positive cells were quantified by flow cytometry using a Cell Lab Quanta SC instrument (Beckman Coulter). The percent neutralization was determined by comparing viral infectivity (denoted as percentage of fluorescent-positive cells) in the presence of serum with the mean infectivity in the absence of serum [24]. Neutralizing antibody positivity was defined as ≥50% neutralization of virus infectivity. Titers are expressed as the reciprocal value of the highest dilution giving ≥50% neutralization of virus growth.

### Epitope-specific antibody assay

Three linear B-cell epitope in HA of 2009 H1N1 and an epitope located in hepatitis C virus envelope protein 2, were synthesized by Biovisualab Inc (Shanghai, China). Peptides were coated onto microplates (1 µg/well) overnight at 4°C. After blocking with BSA buffer, epitope-specific antibody levels in human and mouse sera were assayed as described above.

### Statistical analysis

To estimate the value of the MN titer corresponding to the ELISA titer of anti-2009 HA antibodies, we performed a correlation analysis using linear regression models. Data were analyzed using linear regression and multivariable models, Student' t-test, and estimation of geometric mean titers (GMTs) with confidence intervals and corresponding P values, using the SAS software (ver. 9.1). Data were expressed as mean±standard deviations (SD).

## Results

### High percentage of sera reacted with the HA protein of 2009 pandemic H1N1 virus

**Figure 1:**
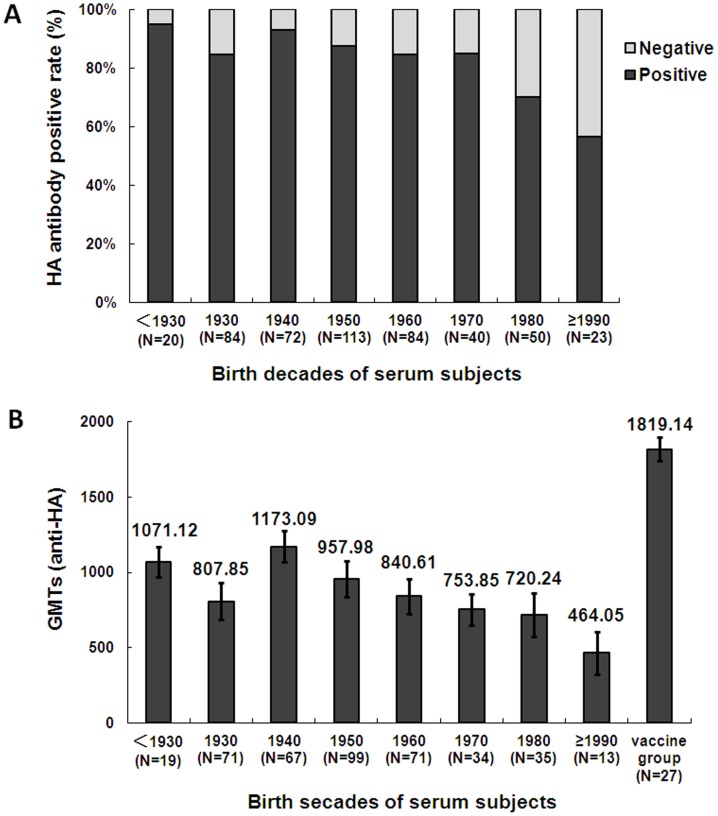
Positive rate and titers of antibody to 2009 HA according to birth decade (1915–2007). A, Proportion of seropositive versus-negative subjects by birth decades. B, Cumulative geometric mean titers (GMT) for all subjects by birth decades. GMTs of 27 young adults immunized with inactivated 2009 H1N1 vaccine were also assessed.

To investigate pre-existing immunity in the general population in Shanghai, 486 serum samples, obtained pre-pandemic, were assessed for an antibody response to 2009 pandemic H1N1 by in-house-developed ELISA. Of the total number of subjects, 84.2% (409/486) possessed serum antibody against the HA of 2009 H1N1 virus. Positivity rates increased from 56.5% of those born after 1990 to 95.0% born before 1930 (Fig. 1A). The GMTs of all positive subjects increased from 464.05±195.63 (subjects born after 1990) to 1173.09±112.62 (born 1940–1990), decreased to 807.85±133.72 (born 1930–1940), then increased again to as high as 1071.12±101.21 (born prior to 1930) (Fig. 1B). All serum samples from 27 volunteers immunized with the inactivated 2009 H1N1 vaccine (collected 1 month after vaccination) were antibody-positive (GMT 1819.14±76.98; Fig. 1B). There was no significant difference in positivity between male and female subjects (male: 210/245; female: 199/241). The serum antibodies were further confirmed using immunofluorescence assay as we recently described [24], among the sera with 2009 HA antibodies positive by ELISA, 87.3% (357/409) samples could react with 293T cells transfected with 2009 HA plasmid, as well as all of the sera from volunteers immunized with the inactivated 2009 H1N1 vaccine, yet none of the negative sera designated as negative control could react with 2009 HA transfected 293 T cells (data not shown). In conclusion, high rates of subjects'sera contained antibody to HA protein of the 2009 pandemic H1N1 virus.

### HI and neutralizing antibody analysis

**Figure 2:**
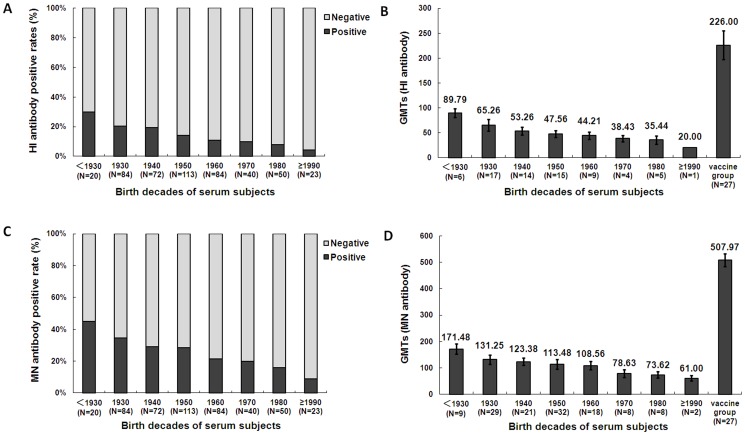
HI and MN antibody positivity rates and titers. A, Proportion of subjects that were HI antibody-positive versus-negative according to age ranges. B, GMTs of HI antibody by age ranges. GMTs of 27 young adults immunized with 2009 H1N1 vaccine were also assessed. C, Proportion of subjects that were neutralizing antibody-positive versus-negative according to age range. D, GMTs of neutralizing antibody by age range. GMTs of 27 young adults immunized with 2009 H1N1 vaccine were also assessed.

Serum antibodies that inhibit HA function of 2009 H1N1 virus were determined by HI and MN assay. Sera showed no reactivity with the HA of 2009 pandemic H1N1 virus in ELISA did not display HI and neutralizing activity against 2009 H1N1 virus, and all HI antibody-positive serum samples showed neutralizing activity (data not shown). Similar to HA antibody profile, HI and MN antibody roughly increased with the age. The overall positive rate of HI antibody was 14.61%. HI antibody positive rate increased from 4.3% of those born after 1990 to 30% of those born before 1930 (Fig. 2A). GMTs of the antibody titers increased from 20 (born after 1990) to 89.79±12.98 (born before 1930) (Fig. 2B). The overall positive rate of MN antibody was as high as 26.5%. Neutralizing antibody positive rate increased from 11.2% of those born after 1990 to 43.5% of those born before 1930 (Fig. 2C). GMTs increased from 61.00±14.14 (born after 1990) to 171.48±13.64 (born before 1930) (Fig. 2D). The HI and MN antibody levels of general population were significantly lower than that of the subjects vaccinated with the inactivated 2009 pandemic H1N1 virus (Fig. 2A–D).

### Correlation of anti-HA IgG titers with neutralizing activity

**Figure 3:**
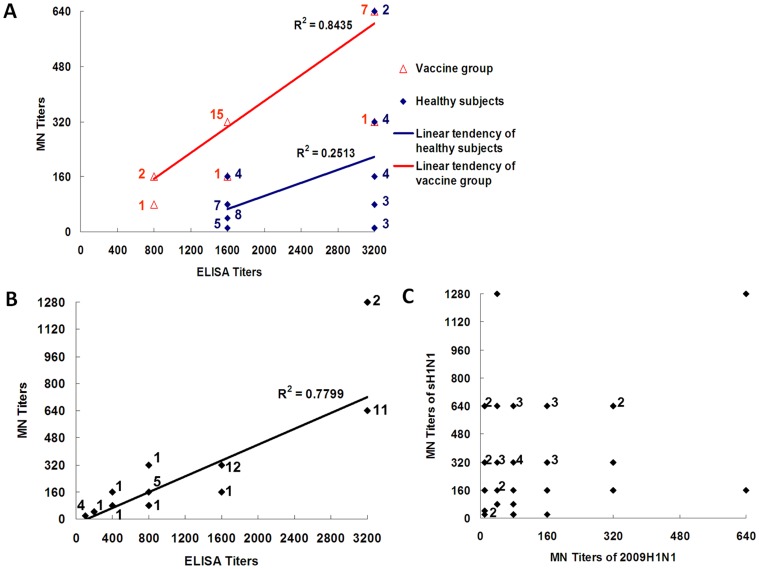
Correlation between IgG titers and neutralizing activity. A, Correlation analysis of anti-2009 HA IgG and MN titers in 40 serum samples with IgG titers ≥1600. For reference, sera from 27 young adults immunized with inactivated 2009 H1N1 vaccine were included. Correlation between IgG and MN titers was calculated by square-coefficient correlation (R2). *N was stated beside data points. B, Correlation analysis of anti-seasonal H1N1 HA and neutralizing antibody titers in the general subjects (n = 40). The correlation between them was analyzed as described above.*N was stated beside data points. C, Correlation of titers of anti-2009 H1N1 and anti-seasonal H1N1 neutralizing antibodies in the general subjects (n = 40). *N was stated beside data points.

To examine the relationship between anti-HA IgG titer and the MN antibody titer, 40 serum samples with high anti-2009 HA IgG titers (GMT≥1,600) were selected for correlation assay. Neutralizing activity did not correspond to the antibody levels of anti-HA. The linear correlation index between IgG and microneutralization antibody titers in general subjects (R^2^_healthy_) was as low as 0.2543, while that of vaccinated subjects (R^2^_vacc_) was 0.8435, suggesting that subjects were exposed to 2009 pandemic H1N1-like viruses (Fig. 3A), while the immunity of vaccinated subjects was elicited by 2009 pandemic H1N1 vaccine (Fig. 3A). The poor correlation between anti-HA titers and neutralizing titers may be explained by that the anti-HA antibodies detected by ELISA reacted with epitopes common between pandemic and seasonal H1N1 viruses.

Serum samples with high titers of anti-HA of 2009 H1N1 virus (n = 40) were further tested for reactivity and MN titer to seasonal human influenza virus H1N1. Sera with high cross-reactive responses to HA of seasonal H1N1 virus also possessed high MN titers to seasonal influenza H1N1 virus (R^2^ = 0.7799; Fig. 3B). We then analyzed the correlation between the MN titers against 2009 pandemic H1N1 and seasonal H1N1 (Fig. 3C), a high MN titer to 2009 pandemic H1N1 did not correlate with a high MN titer to seasonal H1N1, and *vice versa* (Fig. 3C). In conclusion, subjects possessed more specific neutralizing antibodies against seasonal human influenza H1N1 virus than against 2009 pandemic H1N1 virus. These data indicated that subjects had been exposed to infection by seasonal human H1N1 virus more frequently than by a 2009 H1N1-like virus. The data also demonstrated the specificity of the ELISA we developed to detect HA antibodies.

### Strain-specific humoral immune response to seasonal and 2009 pandemic influenza

**Figure 4:**
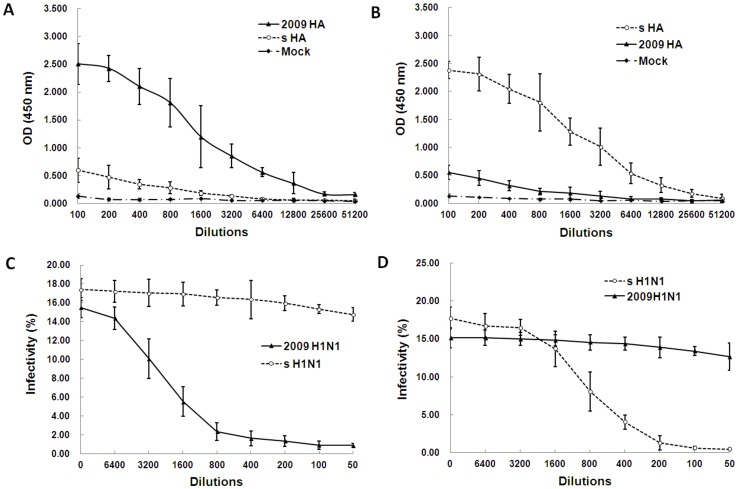
Cross-reactivity and cross-neutralizing activity analysis of antibodies against 2009 pandemic and seasonal H1N1 HAs in mice. Three groups of mice were respectively immunized with 2009 HA, seasonal H1N1 HA or mock plasmid for a total of three times, the sera were collected at two weeks after the last immunization and then used to assay HA antibody and neutralization activity. A, Reactivity of 2009 HA immune sera with 2009 HA and seasonal HA (s HA). B, Reactivity of seasonal influenza HA immune sera with 2009 HA and seasonal HAs. C, Inhibition of 2009 HA immune sera on 2009 pandemic and seasonal H1N1 viruses. D, Inhibition of seasonal H1N1 HA immune sera on seasonal and 2009 pandemic H1N1 viruses.

To examine the cross-reactivity and cross-neutralizing activity of antibodies against the HAs of 2009 pandemic and seasonal influenza viruses, we immunized BALB/c mice with HA expression plasmids and subjected the resulting sera to ELISA. Mice immunized with the 2009 HA-encoding plasmid displayed high reactivity to 2009 pandemic H1N1 HA, and only weak cross-reactivity to seasonal H1N1 HA (Fig. 4A). We then assessed serum microneutralization of the two virus strains. As expected, mice immunized with the 2009 HA-encoding plasmid neutralized the 2009 pandemic H1N1 virus effectively, but had no effect on seasonal influenza H1N1 virus (Fig. 4C). Conversely, mice immunized with a seasonal influenza H1N1 HA-encoding plasmid displayed good reactivity to the HA protein of its ancestor virus, and only weak cross-reactivity with 2009 pandemic H1N1 HA (Fig. 4B). Similarly, sera from immunized mice neutralized the ancestor virus effectively, but had no effect on 2009 pandemic H1N1 virus (Fig. 4D).

### GLFGAIAGFIE is not a valid B cell epitope

**Figure 5:**
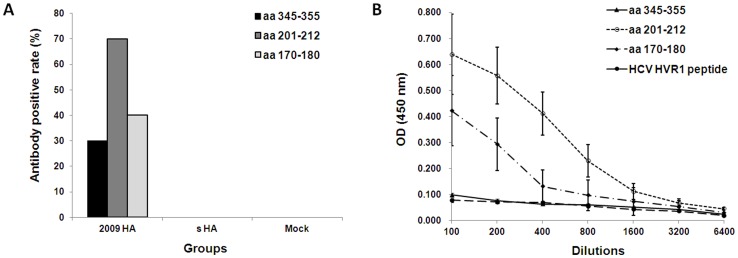
Comfirmation of the conserved linear B cell epitope in H1N1 HA. A, Positive rate of antibody against three peptides in 2009 H1N1 HA in sera of mice immunized with 2009 HA, seasonal HA (s HA) or mock plasmid (n = 10 mice per group). B, Epitope-specific antibody levels in 2009 H1N1 HA DNA immunized mice, HCV HVR1 epitope peptide was used as negative control.

The GLFGAIAGFIE (aa 345–355 in 2009 H1N1 HA) was predicted as the only conserved B cell epitope in HA of 2009 H1N1 and human seasonal H1N1 [14]. To confirm this predicted epitope, synthetic peptide GLFGAIAGFIE, TSADQQSLYQNA (aa 201–212 in 2009 H1N1 HA) and KKGNSYPKLSK (aa 170–180 in 2009 H1N1 HA) were used to detect corresponding antibodies in human or mouse sera. The latter two peptides represent two linear epitopes described by Brownlee and Fodor [26]. A linear epitope peptide GLVGLLTPGAK in hypervariable region 1 (HVR1) of hepatitis C virus envelope protein 2 (E2) was used as negative control. None of the sera from study subjects showed reactivity to peptide aa 345–355. In contrast, among 409 serum samples positive for antibody against 2009 pandemic H1N1 HA, there were 74 and 53 sera positive for antibody against peptide aa 201–212 and aa 170–180 respectively. More specifically, sera from mice received 2009 pandemic H1N1 HA DNA immunization could react with peptide aa 201–212 and aa 170–180, but could not react with peptide aa 345–355 (Fig. 5A and 5B). Sera from mice received seasonal H1N1 HA DNA immunization could not react with any of the peptides (data not shown). Collectively, the GLFGAIAGFIE peptide was not an immunologically valid epitope.

## Discussion

Despite the initial high mortality in Mexico, 2009 pandemic H1N1 virus caused generally mild symptoms, and overall mortality remained at around 0.45% (www.who.int/csr/don/2009_07_06/en). This was not significantly higher than that of seasonal influenza. Pre-existing immunity has been assumed to contribute to the overall low morbidity [13]. However, because the novel pandemic influenza virus contained a unique reassortment of gene segments from both North American and Eurasian swine lineages [27], it was initially supposed that little protective immune memory existed in the general human population. Greenbaum et al. analyzed immune epitopes of recently circulating H1N1 and H3N2 virus strains and found that although eight epitopes were conserved in 2009 pandemic H1N1 virus, only a single B cell epitope was conserved in 2009 HA [7]. This suggested that only low levels of pre-existing neutralizing antibodies were present in the general human population. The Centers for Disease Control and Prevention (Atlanta, GA, USA) reported that among persons >60 years old, 34% had pre-existing, cross-reactive neutralizing antibodies against the new virus [8]. Additionally, Itoh et al. reported that no appreciable neutralizing antibodies against CA04 (A/California/04/09) were detected in individuals born after 1920; however, many of those born before 1918 had these antibodies in high titers [9]. Many serological studies performed on geographically diverse populations also demonstrated pre-existing neutralizing antibodies, with the exception of one report from Singapore [6]–[31]. The authors suggested that pre-existing immunity in elderly subjects had been elicited by infection with 1918-related H1N1 virus.

In the present study, high levels of neutralizing antibodies existed in the Chinese population before the 2009 pandemic H1N1; as many as 84.16% of subjects were seropositive for 2009 HA and 26.5% of sera could neutralize 2009 pandemic virus. The ratios of neutralizing antibody positivity increased with age, and many subjects possessed a high neutralizing antibody titer, consistent with the low morbidity reported in this area. A study performed in Guangxi Province, mainland China, suggested that only 0.3% of subjects possessed low titers of neutralizing antibody, which differed from our findings [6]. Since the neutralizing activity was checked by HI and MN in our study, the differences in population and region/location may be the reason. In fact, human H1N1 viruses circulated in the population from 1918 to 1957 and again from 1977 till now. The classical swine virus HA gene of 2009 pandemic H1N1 is a descendant of the 1918 pandemic virus, and in 1977, H1N1 viruses re-emerged that were genetically and antigenically very closely related to viruses circulating in the 1950s [32]–[35]. Thus all of these circulating viruses may had elicited and/or boosted the neutralizing antibody response to the 2009 pandemic H1N1 virus in subjects of all ages.

On mainland China, due to the extremely high human, swine, and poultry population density, and environmental contamination, influenza viruses might cause undetected infections. The prevalence of antibodies against hepatitis A and E viruses in individuals aged over 40 were approximately 100% and 40%, respectively; the aforementioned factors were believed to be the major reasons for such high rates [1]. The positive rate of anti-HA and neutralizing antibody against 2009 pandemic H1N1 virus presented here suggested that many of the subjects in this study had been exposed to antigenically related viruses. Our data also suggest that many subjects had been exposed repeatedly to unidentified viruses, including the virus strains with hemagglutinins related with 2009 pandemic and seasonal H1N1 strains antigenically, thus inducing immunity to a particular influenza strain. The unexpectedly robust immune response of Chinese subjects vaccinated with inactivated 2009 pandemic H1N1 support this view [36].

There was an obvious discrepancy of the proportions: the total anti-HA antibodies rate (84.2%), MN antibodies rate (26.5%) and HI antibodies rate (14.61%), but all the MN positive sera were also anti-HA positive, and all the HI positive sera were MN positive. Compared with the subjects immunized 2009 H1N1 vaccines, the correlation between anti-HA titers and MN titers of the general population was relatively lower. It may be explained by different epitopes in HA involved in immune response. In this regard, major anti-HA antibodies may target the conserved stalk region of HA, while the neutralizing antibodies were against the hypervariable globular head of HA. On the other hand, as described above, our data may also indicate that the immunity against 2009 H1N1 in general Shanghainese was derived from complicated circulating influenza viruses, this possibility was supported by studies from Taiwan and France [31], [37]. Additionally, a monoclonal antibody recognizing a neutralizing epitope common among H1, H2, H5, and H6 was demonstrated to protect mice from a lethal challenge with various H5N1 and 2009 H1N1 [38]. Consistent with these observations, more recently, it was reported that neutralizing antibodies induced by 2009 pandemic H1N1 infection were broadly reactive against divergent H1N1 and H5N1 influenza strains [39].

A 2009 H1N1 isolate was more pathogenic in mice and ferrets with no previous exposure to influenza, indicating that pre-existing cross-reactive antibodies might offer partial protection against novel influenza strains [40]. Several studies demonstrated that the seasonal influenza vaccines did not elicit cross-reactive neutralizing antibodies against the 2009 pandemic H1N1 virus in subjects of any age [41]–[43]. In this study, we investigated this by assessing the immune response of mice immunized using HA-encoding plasmids. The results showed that only very low level of cross-reactive HA antibody was induced and the immune sera exerted little cross-neutralization. Additionally, the only predicted conserved linear epitope GLFGAIAGFIE in HAs among diverse H1N1 strains was demonstrated as invalid, suggesting the rarity of cross protective epitopes in 2009 H1N1 and seasonal H1N1 virus. Together with the observation that natural infection with some seasonal H1N1 viruses may induce a cross-reactive antibody response to the 2009 H1N1 virus, it is possible that cross-neutralization epitopes exist among 2009 H1N1 and other influenza virus strains [31], [37]. Whether immune responses to different influenza viruses can amplify each other, and precisely how many strain-specific humoral immune responses exist in Shanghainese population, is remained to be investigated in future studies.

## References

[1] Cao J, Wang Y, Song H, Meng Q, Sheng L, et al (2009) Hepatitis A outbreaks in China during 2006: application of molecular epidemiology. Hepatol Int 3: 356–363. 19669361 10.1007/s12072-008-9116-8 PMC2716766

[2] Chowell G, Bertozzi SM, Colchero MA, Lopez-Gatell H, Alpuche-Aranda C, et al (2009) Severe respiratory disease concurrent with the circulation of H1N1 influenza. N Engl J Med 361: 674–679. 19564633 10.1056/NEJMoa0904023

[3] Mathews JD, McCaw CT, McVernon J, McBryde ES, McCaw JM (2007) A biological model for influenza transmission: pandemic planning implications of asymptomatic infection and immunity. PLoS One 2: e1220. 18043733 10.1371/journal.pone.0001220 PMC2080757

[4] Mathews JD, McBryde ES, McVernon J, Pallaghy PK, McCaw JM (2010) Prior immunity helps to explain wave-like behaviour of pandemic influenza in 1918–9. BMC Infect Dis 10: 128. 20497585 10.1186/1471-2334-10-128 PMC2891754

[5] Scalera NM, Mossad SB (2009) The first pandemic of the 21st century: a review of the 2009 pandemic variant influenza A (H1N1) virus. Postgrad Med 121: 43–47. 19820273 10.3810/pgm.2009.09.2051

[6] Chen H, Wang Y, Liu W, Zhang J, Dong B, et al (2009) Serologic survey of pandemic (H1N1) 2009 virus, Guangxi Province, China. Emerg Infect Dis 15: 1849–1850. 19891883 10.3201/eid1511.090868 PMC2857250

[7] Greenbaum JA, Kotturi MF, Kim Y, Oseroff C, Vaughan K, et al (2009) Pre-existing immunity against swine-origin H1N1 influenza viruses in the general human population. Proc Natl Acad Sci USA 106: 20365–20370. 19918065 10.1073/pnas.0911580106 PMC2777968

[8] Hancock K, Veguilla V, Lu X, Zhong W, Butler EN, et al (2009) Cross-reactive antibody responses to the 2009 pandemic H1N1 influenza virus. N Engl J Med 361: 1945–1952. 19745214 10.1056/NEJMoa0906453

[9] ItohY, Shinya K, Kiso M, Watanabe T, Sakoda Y, et al (2009) In vitro and in vivo characterization of new swine-origin H1N1 influenza viruses. Nature 460: 1021–1025. 19672242 10.1038/nature08260 PMC2748827

[10] Ikonen N, Strengell M, Kinnunen L, Osterlund P, Pirhonen J, et al (2010) High frequency of cross-reacting antibodies against 2009 pandemic influenza A(H1N1) virus among the elderly in Finland. Euro Surveill 15 pii: 19478. 20144443

[11] Kwan-Gett TS, Baer A, Duchin JS (2009) Spring 2009 H1N1 influenza outbreak in King County, Washington. Disaster Med Public Health Prep 3 Suppl 2S109–116. 19952883 10.1097/DMP.0b013e3181c6b818

[12] Miller E, Hoschler K, Hardelid P, Stanford E, Andrews N, et al (2010) Incidence of 2009 pandemic influenza A H1N1 infection in England: a cross-sectional serological study. Lancet 375: 1100–1108. 20096450 10.1016/S0140-6736(09)62126-7

[13] Xing Z, Cardona CJ (2009) Preexisting immunity to pandemic (H1N1) 2009. Emerg Infect Dis 15: 1847–1849. 19891882 10.3201/eid1511.090685 PMC2857244

[14] Garten RJ, Davis CT, Russell CA, Shu B, Lindstrom S, et al (2009) Antigenic and genetic characteristics of swine-origin 2009 A(H1N1) influenza viruses circulating in humans. Science 325: 197–201. 19465683 10.1126/science.1176225 PMC3250984

[15] Zhang Y, Li X, Zhang F, Wu J, Tan W, et al (2009) Hemagglutinin and neuraminidase matching patterns of two influenza A virus strains related to the 1918 and 2009 global pandemics. Biochem Biophys Res Commun 387: 405–408. 19615337 10.1016/j.bbrc.2009.07.040 PMC7092934

[16] Democratis J, Pareek M, Stephenson I (2006) Use of neuraminidase inhibitors to combat pandemic influenza. J Antimicrob Chemother 58: 911–915. 16956904 10.1093/jac/dkl376

[17] Gerdil C (2003) The annual production cycle for influenza vaccine. Vaccine 21: 1776–1779. 12686093 10.1016/s0264-410x(03)00071-9

[18] Subbarao K (1999) Influenza vaccines: present and future. Adv. Virus Res 54: 349–373. 10.1016/s0065-3527(08)60371-1 10547679

[19] Du N, Zhou J, Lin X, Zhang Y, Yang X, et al (2010) Differential activation of NK cells by influenza A pseudotype H5N1 and 1918 and 2009 pandemic H1N1 viruses. J Virol 84: 7822–7831. 20484512 10.1128/JVI.00069-10 PMC2897595

[20] Lin X, Zhou J, Zhang Y, Wu J, Zhang F, et al (2009) Oseltamivir boosts 2009 H1N1 virus infectivity in vitro. Biochem Biophys Res Commun 390: 1305–1308. 19879239 10.1016/j.bbrc.2009.10.142

[21] Liu Y, Liu X, Fang J, Shen X, Chen W, et al (2010) Characterization of antibodies specific for hemagglutinin and neuraminidase proteins of the 1918 and 2009 pandemic H1N1 viruses. Vaccine 29: 183–190. 21055499 10.1016/j.vaccine.2010.10.059

[22] Wu J, Zhang F, Wang M, Xu C, Song J, et al (2010) Characterization of neuraminidases from the highly pathogenic avian H5N1 and 2009 pandemic H1N1 influenza A viruses. PLoS One 5: e15825. 21209916 10.1371/journal.pone.0015825 PMC3012118

[23] Zhang Y, Lin X, Wang G, Zhou J, Lu J, et al (2010) Neuraminidase and hemagglutinin matching patterns of a highly pathogenic avian and two pandemic H1N1 influenza A viruses. PLoS One 5: e9167. 20161801 10.1371/journal.pone.0009167 PMC2820099

[24] Fang J, Chen Z, Liu X, Li H, Wang J, Shen X, Chen W, et al (2011) Immunization with a low dose of hemagglutinin-encoding plasmid protects against 2009 H1N1 pandemic influenza virus in mice. J Virol Methods 173(2): 314–319. 21392537 10.1016/j.jviromet.2011.03.001

[25] Reed LJ, Muench H (1938) A simple method of estimating fifty percent endpoints. Am J Epidemiol 27: 493–497.

[26] Brownlee GG, Fodor E (2001) The predicted antigenicity of the haemagglutinin of the 1918 Spanish influenza pandemic suggests an avian origin. Philos Trans R Soc Lond B Biol Sci 356(1416): 1871–1876. 11779386 10.1098/rstb.2001.1001 PMC1088563

[27] Smith DJ, Lapedes AS, de Jong JC, Bestebroer TM, Rimmelzwaan GF, et al (2004) Mapping the antigenic and genetic evolution of influenza virus. Science 305: 371–376. 15218094 10.1126/science.1097211

[28] Tang JW, Tambyah PA, Wilder-Smith A, Puong KY, Shaw R, et al (2010) Cross-reactive antibodies to pandemic (H1N1) 2009 virus, Singapore. Emerg Infect Dis 16: 874–876. 20409391 10.3201/eid1605.091678 PMC2954004

[29] Xu R, Ekiert DC, Krause JC, Hai R, Crowe JR, et al (2010) Structural basis of preexisting immunity to the 2009 H1N1 pandemic influenza virus. Science 328: 357–360. 20339031 10.1126/science.1186430 PMC2897825

[30] Rizzo C, Rota MC, Bella A, Alfonsi V, Declich S, et al (2010) Cross-reactive antibody responses to the 2009 A/H1N1v influenza virus in the Italian population in the pre-pandemic period. Vaccine 28(20): 3558–3562. 20307592 10.1016/j.vaccine.2010.03.006

[31] Chi CY, Liu CC, Lin CC, Wang HC, Cheng YT, et al (2010) Preexisting antibody response against 2009 pandemic influenza H1N1 viruses in the Taiwanese population. Clin Vaccine Immunol 17(12): 1958–1962. 20876823 10.1128/CVI.00212-10 PMC3008195

[32] Dowdle WR, Hattwick MA (1977) Swine influenza virus infections in humans. J Infect Dis 136 Suppl:S386–389 10.1093/infdis/136.supplement_3.s386 342616

[33] Olsen CW (2002) The emergence of novel swine influenza viruses in North America. Virus Res 85: 199–210. 12034486 10.1016/s0168-1702(02)00027-8

[34] Taubenberger JK, Hultin JV, Morens DM (2007) Discovery and characterization of the 1918 pandemic influenza virus in historical context. Antivir Ther 12(4 Pt B): 581–591. 17944266 PMC2391305

[35] Tumpey TM, Basler CF, Aguilar PV, Zeng H, Solórzano A, et al (2005) Characterization of the reconstructed 1918 Spanish influenza pandemic virus. Science 310: 77–80. 16210530 10.1126/science.1119392

[36] Liang XF, Wang HQ, Wang JZ, Fang HH, Wu J, et al (2010) Safety and immunogenicity of 2009 pandemic influenza A H1N1 vaccines in China: a multicentre, double-blind, randomised, placebo-controlled trial. Lancet 375(9708): 56–66. 20018364 10.1016/S0140-6736(09)62003-1

[37] Lemaitre M, Leruez-Ville M, De Lamballerie XN, Salez N, Garrone P, et al (2010) Seasonal H1N1 2007 influenza virus infection is associated with elevated pre-exposure antibody titers to the 2009 pandemic influenza A (H1N1) virus. Clin Microbiol Infect 17(5): 732–737. 20731679 10.1111/j.1469-0691.2010.03352.x

[38] Sakabe S, Iwatsuki-Horimoto K, Horimoto T, Nidom CA, Le Mt, et al (2010) A cross-reactive neutralizing monoclonal antibody protects mice from H5N1 and pandemic (H1N1) 2009 virus infection. Antiviral Res 88(3): 249–255. 20849879 10.1016/j.antiviral.2010.09.007 PMC2991629

[39] Wrammert J, Koutsonanos D, Li GM, Edupuganti S, Sui J, et al (2011) Broadly cross-reactive antibodies dominate the human B cell response against 2009 pandemic H1N1 influenza virus infection. J Exp Med 208(1): 181–193. 21220454 10.1084/jem.20101352 PMC3023136

[40] Maines TR, Jayaraman A, Belser JA, Wadford DA, Pappas C, et al (2009) Transmission and pathogenesis of swine-origin 2009 A(H1N1) influenza viruses in ferrets and mice. Science 325: 484–487. 19574347 10.1126/science.1177238 PMC2953552

[41] Pascua PN, Song MS, Lee JH, Park KJ, Kwon HI, et al (2009) Evaluation of the efficacy and cross-protectivity of recent human and swine vaccines against the pandemic (H1N1) 2009 virus infection. PLoS One 4: e8431. 20037716 10.1371/journal.pone.0008431 PMC2793524

[42] Centers for Disease Control and Prevention (2009) Serum cross-reactive antibody response to a novel influenza A (H1N1) virus after vaccination with seasonal influenza vaccine. MMWR Morb Mortal Wkly Rep 58(19) 521–524. 19478718

[43] Centers for Disease Control and Prevention (2009) Effectiveness of 2008–09 trivalent influenza vaccine against 2009 pandemic influenza A (H1N1)-United States, May-June 2009. MMWR Morb Mortal Wkly Rep 58: 1241–1245. 19910912

